# Translating ‘dementia friends’ programme to undergraduate medical and nursing practice: a qualitative exploration

**DOI:** 10.1186/s12909-023-04561-1

**Published:** 2023-08-07

**Authors:** Stephanie Craig, Christine Brown Wilson, Gary Mitchell

**Affiliations:** School of Nursing and Midwifery, Queen’s University Belfast, Belfast, Northern Ireland

**Keywords:** Dementia, Alzheimer’s Disease, Dementia Friends, Medical Education, Nursing Education

## Abstract

**Introduction:**

Dementia awareness is a key priority of medical and nursing pre-registration education. The ‘dementia friends’ programme is an internationally recognised and accredited dementia awareness workshop that is led by a trained facilitator. While this programme has been associated with positive outcomes, few studies have examined how medical and nursing students apply their learning in practice after the workshop. The aim of his study was to explore how nursing and medical students apply the dementia friend’s programme into practice when caring for people living with dementia.

**Methods:**

Seven focus-group interviews were conducted with 36 nursing students and 14 medical students at one university in Northern Ireland (n = 50), following ‘the dementia friends programme. Interview guides were co-designed alongside people living with dementia. Interviews were audio-recorded, transcribed verbatim and analysed using thematic analysis. Ethical approval was granted for this study.

**Results:**

Four themes emerged: *‘reframing dementia’*, which highlighted how the education had enabled students to actively empower and support people living with dementia in practice; *‘dementia friendly design’*, which focused on how students had modified their clinical environments when providing care for people living with dementia, *‘creative communication’*, which considered how students had used their education to adapt their verbal and non-verbal communication with people living with dementia and *‘realities of advanced dementia’* which contemplated how students believed their dementia education could be improved within their current curriculum.

**Discussion:**

The Dementia Friends programme has actively supported nursing and medical students to improve the lives of people with dementia in their care through environmental adaptions and creative approaches to communication. This study provides an evidence base that supports the provision of ‘a dementia friends programme to healthcare professional students. The study also highlights how this education can actively influence how nursing and medical students support people living with dementia in their practice in the months and years after education.

**Supplementary Information:**

The online version contains supplementary material available at 10.1186/s12909-023-04561-1.

## Background

Raising dementia awareness is a priority policy issue in the United Kingdom and internationally [1]. Dementia is a clinical illness marked by a significant decline in cognitive and emotional abilities [2] that is severe enough to impede everyday activities and quality of life beyond what is normally expected with biological ageing [3]. This disease affects more than 57 million people worldwide [4], with over 10 million new cases diagnosed each year [5]. Individuals impacted by the syndrome might experience a wide range of stigma and discrimination [6], which is typically related to a general misunderstanding of the disease. Therefore, dementia education, awareness and intervention are vital. To combat the associated stigmas the Alzheimer’s Society developed the ‘Dementia Friends’ Programme in the United Kingdom (UK) in 2013 and within two years, 2.8 million people had received this educational programme [1]. This was one of the largest social action initiatives to alter people’s perceptions of dementia aiming to ‘change the way we think, act and talk about dementia’ [7]. The ‘dementia friends’ programme [8] combines theory and practice to help people in their communities who are living with dementia. The ‘dementia friends’ programme involves a facilitator hosting a face-to-face workshop, of approximately 12–20 people, about living with dementia. The length of the session can vary from 90 minutes to 2-hour sessions, and the content covers key messages about dementia, engaging activities and discussions, with the opportunity for attendees to commit to a dementia-friendly action [9].

The programme provides learners with the opportunity to support people with dementia to live well, both inside a care setting and outside formal care settings, for example in their local communities. This is because the ‘dementia friends programme is closely linked to the development of “Dementia Friendly Communities” (DFCs) [10]. A DFC is defined as a place or culture where individuals with dementia are recognised, valued, supported, and confident in their ability to contribute to society [11]. DFCs are key in helping people with dementia to live well and stay active members of their communities hence the need for future healthcare professionals to have the knowledge to empower people with dementia. Usually, people involved within a DFC will understand dementia and know about ways to empower to live well with the condition. Providing this programme to undergraduate nursing and medical students may help future healthcare professionals to role-model best behaviours in relation to supporting the development and sustainability of DFCs in the future. One of the key areas of action for developing DFCs is Heath and Social Care, this includes acute and community care settings. Therefore, providing the Alzheimer’s Society Dementia Friends programme to nursing and medical students will contribute to the development of DFCs in the future as it is more than dementia education it is about enhancing society too.

Within the higher education institution of the authors, dementia is comprehensively integrated into the nursing and medical undergraduate programmes. However, prior to the undertaking of this study, the emphasis in both programs primarily revolved around the biomedical aspects of care, such as the aetiology, pathophysiology, clinical manifestations of dementia diseases, nursing and medical interventions, pharmacology, non-pharmacological treatments, palliative care, and more. While these topics adequately fulfilled the educational requirements set forth by the Nursing and Midwifery Council (NMC) and the General Medical Council (GMC), a significant gap persisted within both programs. This gap was characterised by the absence of perspectives from individuals living with dementia, as well as limited consideration given to the personhood of individuals affected by the illness and the practical challenges, they encounter in their lives beyond the confines of the hospital setting. Consequently, a supplementary educational initiative known as the “dementia friends” program was introduced to shed light on the profound impact of increased awareness and understanding of dementia on improving practical care practices. The incorporation of this aspect of education would therefore be more likely to provide undergraduate medical and nursing students with holistic education about dementia.

Holistic dementia education is also considered to be a global priority in health professions education [12–15]. For example, medical and pharmacy students in Malaysia [14] highlighted the necessity for students to understand dementia from the perspective of the person, while nursing and medical students in China [15] identified the importance of an interdisciplinary approach to dementia education to ensure students have sufficient knowledge, attitudes, and experiences in the treatment of persons with dementia and in America, they have positively evaluated the impact of dementia friends sessions which significantly increased the knowledge and positive attitudes of health professional students towards those living with dementia after a one- hour information session [16].

In practice nurses and doctors, together with health professions students, play a critical role in supporting the person living with dementia from pre-diagnosis to advance stages of care such as end-of-life [17–19]. The ‘dementia friends’ initiative is part of a global initiative that promotes awareness and fosters empathy in learners [7]. Despite its widespread and international application [20], few studies have examined how the Alzheimer’s’ Society ‘dementia friends’ programme is applied by medical and nursing students as part of their learning journey. It is a global recommendation that healthcare professionals (and students) know more about dementia and have better dementia awareness [21]. Therefore, the aim of this research is to explore how both nursing and medical students apply the dementia friend’s programme into practice when caring for people living with dementia.

## Methods

### Design, setting, population

A qualitative methodology was used as it is equipped to provide an in-depth exploration of how student nurses and student doctors translate their knowledge from the dementia friends’ programme into their practice during clinical placement. Data were collected using focus groups enabling students to talk about their experiences about the dementia friends’ programme. Using multiple focus groups has the potential for researchers to identify the similarities and differences of experience within and across groups [22].

The study was conducted using non-probability convenience sampling of year two/three undergraduate adult, mental health and learning disability nursing students and year four/five undergraduate medical students (n = 1050) from Queen’s University Belfast (QUB) in Northern Ireland. Participants were eligible if they had attended the ‘dementia friends’ programme in the previous 12–24 months of their programme. At the time of this study, the ‘Dementia Friends’ programme was provided to nursing students in year one of their programme and to medical students in year three of their programme.

### Intervention

In this study, the ‘dementia friends’ programme was delivered by an Alzheimer’s Society trained facilitator from Northern Ireland. In all cases, this was a facilitator that had completed a two-day facilitator course with the Alzheimer’s Society and had an annual peer-review of their delivery in the preceding twelve months. The sessions were timetabled for all medical and nursing students at the home author’s institution. In this study, delivery was via a face-to-face workshop and all sessions lasted between 90 minutes to 120 minutes. The ’dementia friends’ workshop was originally co-designed by people living with dementia and accredited by the Alzheimer’s Society. It uses the methods of face-to-face teaching, facilitation of small group activities, individual reflection and media footage. On completion of the learning, all attendees receive a certificate of completion and a pin-badge that reads “dementia friend”. The programme covers: brain anatomy, how people living with dementia experience the environment, experience memory loss and support that can be provided to help people live well with dementia [23]. The programme is interactive enabling participants to engage in discussion throughout the session.

The workshop, co-designed by people living with dementia, uses the methods of face-to-face teaching, facilitation of small group activities, individual reflection and media footage. The free dementia friend workshop is led by Alzheimer Society Community Champions and works best with tutorial-style training. Everyone who completes the workshop is given a certificate of completion and a pin-badge that reads “dementia friend”.

### Ethics

This study received ethical approval by Queen’s University Belfast, Faculty of Medical, Health and Life Sciences Research Ethics Committee in September 2019 (Ref: GMitchell.SREC_19_V2). Written informed consent was obtained from all who participated in this study. All methods were performed in accordance with the Declaration of Helsinki. The study took place between January 2020-January 2021.

### Consent

Eligible nursing and medical students were contacted via email, by a university administrator not associated with the project, to ask if they would be interested in participating in the focus groups. Potential participants were contacted a total of three times over a six-week period, at two-week intervals. Participants interested in attending the focus group contacted the project lead (SC) who sent an information sheet and an Eventbrite link to sign-up to one of seven pre-scheduled focus group interview slots. If participants had requested to receive information about the study but then did not respond to this information, the project lead was permitted to send one further email to serve as a reminder and invite any study questions.

Student participants were therefore responsible for self-enrolling in focus group interviews, and they were made aware they were under no obligation to attend focus groups and any participation would not positively or negatively impact upon their course grade or progression on their programme of study. Participants were permitted to withdraw from the study at any stage, without giving any reason and information about these processes was provided within the study information sheet. Student participants were required to complete an online informed consent at least 48 h prior to the focus group session they signed up to and then sign a written informed consent form on the day of the focus group prior to data collection. Students who participated in the focus-group received refreshments during the interview. No other incentives were offered.

### Data collection

Data were collected via seven face-to-face focus groups from separate disciplines which were facilitated by two members of the research team (SC & GM). All students who participated in focus groups had received the Alzheimer’s Society’s dementia friends’ programme as part of their course at least 12 months prior. Thirty-six participants were nursing students and fourteen were medical students. The duration of focus group interviews was between 40 and 55 min. The research team (SC, CBW & GM) co-designed the interview guides (Supplementary file 1) alongside four people living with dementia and two charity advocates from a local charity called Dementia NI. The interview guide was co-designed following a face-to-face meeting at Queen’s University Belfast in November 2019. During the meeting, people with dementia told the research team about the questions they would want to ask nursing and medical students after receiving the ‘dementia friends’ education. The two charity advocates, who already had an established relationship with the four people living with dementia, supported the articulation of the priorities for data collection, while the research team documented the questions and reflected these back in real-time. Co-design was therefore a key aspect of the development of the interview guides, as co-designing with people living with dementia who are the experts in their care can uncover service user needs and preferences which have the potential to be overlooked by researchers during the research process [24].

### Data analysis

All data were audio-recorded and fully transcribed verbatim (SC). Thematic analysis was undertaken using Braun and Clark’s [25] six-stage framework: familiarisation with data, generating initial codes, searching for themes, reviewing themes, defining themes, and producing the report. Two members of the team (SC & GM) read and re-read the data independently to generate initial codes. Once the initial codes were agreed upon, these were formed into themes by the same members and reviewed by the other members of the team (CBW).

### Trustworthiness

The four criteria of credibility, transferability, dependability, and confirmability [26] were used to improve the trustworthiness of this research data. The raw and thematic data were made available to the participants for review. Throughout the research process, meticulous record-keeping was maintained. Regular team meetings created an audit trail.

Credibility assesses whether the research conclusions accurately reflect the participants original ideas [26]. In relation to this study, all participants received a brief summary of the findings generated through their participation in order to check that the researcher’s summary accorded with that of the focus group they participated in. Korsten’s and Moser [27] define dependability as the consistency of results over time. Lincoln and Guba [26] encourage researchers to keep good records of all meetings and decisions taken about the research. All meetings between the research team were dated and recorded in case they needed to be viewed in the future. Confirmability is similar to dependability but is achieved through openness in the research process [26] and the ability to produce an audit trail if called upon. Clear records were maintained throughout this research. Finally, transferability refers to the degree to which the research findings can be applied to a variety of contexts or settings. This was achieved through thick description of the dementia friends’ intervention. This enables readers of this research to judge whether the findings can be applied to their setting.

## Results

50 students (36 nursing and 14 medical) from one university in Northern Ireland participated in seven focus groups which were discipline specific. Due to the timetabling of classes, it was not possible to mix focus groups.

Four themes emerged in relation to this study:

1. *Reframing Dementia*: how the education enabled students to actively empower and support people living with dementia in practice.
2. *Dementia Friendly Design*: how students modified their clinical environments when providing care for people living with Dementia.
3. *Creative communication*: how students used their education to adapt their verbal and non-verbal communication with people living with dementia.
4. *Realities of Advanced Dementia*: how students believed their dementia education could be improved within their current curriculum.

### Reframing dementia

The most common theme reported by participants was about how the ‘dementia friends’ programme helped support student nurses and student doctors to have a better understanding of the dementia diseases. Participants valued learning about the social aspects of dementia that were provided in this educational programme as noted in the excerpt below.

*“It wasn’t what I expected, but in a good way, I was thinking anatomy and physiology of the brain or the half-life of Rivastigmine [Cognitive Enhancing Medication] or whatever. Right? But when we got to talking about things like driving or cooking or managing money, I was a bit dumbfounded. I guess I hadn’t really thought of the impact of all that” [FG6, P9, Year 5 Medical Student].*

Medical and nursing students detailed how they translated their knowledge from the ‘dementia friends’ programme to develop a more holistic understanding with their patients in care settings. Examples of this included, greater acknowledgement about how people with dementia could feel disempowered when they could no longer work, manage their own money or make decisions about their own life.

*“Maybe it is just me, but the training [dementia friends] taught me to be more accepting and less dismissive. So, when someone [living with dementia] says they are sad because they can no longer drive, we can take a bit of a step back, listen and empathise. A little bit of empathy, kindness and understanding goes a long way I find” [FG2, P4, Year 2 Nursing Student].*

Greater awareness and understanding of the social challenges people with dementia may face acted as a catalyst for many nursing and medical participants to act. Participants discussed how their knowledge and understanding from the ‘dementia friends’ programme helped them to actively support people with dementia both in care and in society as detailed in the excerpts below.

*“One of the things I did [because of the dementia friends programme] was to support people in the nursing home…in the end stages I mean, to make decisions. Not big ones, but like, those little ones they could still make – what they want to eat, what they want to wear, what time they want to get up at. A sort of ‘doing with’ not ‘doing for’ mentality” [FG4, P5, Year 3 Nursing Student].*

*“The training takes you outside of the ward, it’s a bit like first aid in some ways. You learn about choking, resuscitation and things so you can deal with it if you come across it outside [non-hospital setting]. Well, dementia friends is a bit like that too – you come across someone with dementia struggling to count their money, or not able to find their way in a supermarket – then you can do something to help” [FG7, P4, Year 4 Medical Student].*

Overall, this theme demonstrated how medical and nursing students collectively translated their knowledge about dementia awareness, both in care and social setting. It also demonstrates, how this knowledge provided healthcare students with the confidence to try to make a difference when they encountered people with dementia.

### Dementia Friendly Design

A key focus of the ‘dementia friends programme was around dementia friendly design. Particularly, the ways in which an environment could either facilitate or prohibit people with dementia to live well. This theme illustrates how nursing and medical students helped to modify environments to improve clinical outcomes for people living with dementia. With regards to participant knowledge, after undertaking the ‘dementia friends’ programme, nursing and medical students were more aware of the importance of environmental modifications and their purposes. Prior to receiving this education, nursing and medical students shared disparity about their knowledge in this area.

*“I did know that the signage on doors and stuff was for wayfinding – yes. But before the training [dementia friends], I didn’t know about all the contrasting of colours, you know the toilet seat being bright blue so that someone could see it, or the big high bowls that some people eat out of so that someone knows when their spoon reaches the end of the bowl and whatever. So, it kind of gave me that information” [FG1, P2, Nursing Student Year 2].*

Greater awareness of the environment supported many medical and nursing students to make modifications in practice to enhance quality of life. For example, the removal of mirrors that could further cause confusion to people with dementia, the reduction of loud noise or indeed consideration about the use of patterns were all explicitly noted by multiple participants.

*“I have a great example about this. The home [nursing home] was getting refurbished and they had some carpet samples. We were all talking about it in the tearoom at lunch and I just happened to mention the scenario from Dementia Friends – the person [with dementia] who thought the patterned carpet was a pond with fish…they were like, ‘what?’ and I was like, yeah and told them the story [laughs]. They went for a plain one [carpet] after!” [FG5, P8, Nursing Student Year 2].*

*“I was the opposite to what everyone was saying [about removing potential distressing environmental items]. But I did make sure the person had their memory boxes or photos of family or their handbag or anything personal you know? So, I think that means the person knows their stuff and so the environment is a bit more comforting” [FG5, P5, Nursing Student Year 2].*

While dementia friendly design was something that nursing students appeared to be actively involved in, there was sometimes less assertion from medical students. Examples from medical students appeared to mainly focus on smaller types of environmental factors, such as ensuring water was close to the person with dementia, ensuring they could reach their buzzer or keeping bed rails down to avoid entrapment. In most cases, environmental modifications described by student doctors related to medical practice, for example nutrition or restrictive practice as noted.

*“Well, the nurses are on another level with that one [in reference to actively modifying the environment]. I think as doctors, we are a bit less confident maybe in doing things like that [making environmental changes] …something to work on now we have knowledge” [FG6, P5, Fifth Year Medical Student].*

Therefore, the dementia friends’ programme supported medical, and particularly nursing, students to actively consider how they could improve the environment people with dementia were receiving care in. Participants demonstrated an enhanced awareness of how small environmental changes had the potential to improve quality of life for people living with dementia.

### Creative Communication

The ‘dementia friends’ programme also supported nursing and medical students to employ more creative strategies to enhance their communication with people living with dementia and their caregivers. One aspect that was reflected upon by many participants was the ‘bookcase analogy’ which was taught as part of the dementia friends programme. The bookcase analogy describes how the brain is like a bookshelf, and on the bookshelf are memories with the most recent at the top. As people with dementia progress in their illness, the bookcase gets shaken and recent memories become displaced or fall out while older memories can remain in act.

*“Personally, I hated the bookcase analogy. As a trainee studying medicine, I thought this has absolutely no scientific bearing and it actually would make me more likely to distrust other elements of the course” [FG7, P10, Fourth Year Medical Student].*

*“I liked the bookcase analogy. I don’t think it is meant to be used as a basis for science, but I think it helps us [nurses] explain to scared family members about what is going on, in layman terms” [FG2, P1, Third Year Nursing Student].*

While the bookcase analogy was divisive, there was shared agreement between participants that the ‘dementia friends’ programme supported participants to consider more meaningful modes of communication. The use of music therapy, doll therapy, aromatherapy and garden therapy were all explicitly referenced by participants as ways in which they communicated with people with dementia when needed.

*“I used the doll. The patient [person with dementia] wouldn’t respond to anyone, but when I picked up the doll and started talking to the both of them [patient and the doll], we got verbal responses to the questions we were asking” [FG7, P6, Fourth Year Medical Student].*

*“In the nursing home it is like a musical because I am always singing. It is one of the ways they [people with dementia] respond to me. So, not even like having long conversations, but getting them to smile, clap hands or whatever” [FG3, P3, Second Year Nursing Student].*

Participants in this study also talked about their use of supportive communication aids, such as life history documentation, and how this could be used to develop a rapport with people with dementia. Many participants discussed how the ‘dementia friends’ programme enhanced their appreciation of the value of non-verbal communication and how body language and tone were usually more important than the spoken word.

A key point noted in this theme was the value of interdisciplinary communication with colleagues. An important part of the ‘dementia friends’ programme centres on the importance of non-stigmatising language, for example referring to people, not patients or refraining from using terms like ‘demented’ or ‘sufferer’. Medical and nursing students felt that, as dementia friends, they needed to role model best practice and that needed to be evident by the words they spoke and wrote as noted below.

*“It’s hard to call it out because I don’t think anyone means any harm [using labelling language], so I figure, if I just ‘do me’, then maybe that will rub off – you know what I am saying? Not demented, its dementia, not elderly, it’s older, not patient, person and all the rest of it” [FG7, P7, Fourth Year Medical Student].*

Overall, this theme illuminated how the dementia friends’ programme can positively impact the verbal and non-verbal communication processes that nursing and medical students used with people living with dementia, their family members and their interdisciplinary colleagues.

### Realities of Advanced Dementia

The fourth theme to emerge from the data was less prominent compared to the others, but nonetheless it is noteworthy as it was referenced by five out of the seven focus groups. This theme referenced to a consistent recommendation from student doctors and nurses about current dementia friends programme. This recommendation was explicitly discussed across five of the seven focus groups and there was consensus that, while it is positive that the ‘dementia friends’ programme focuses on living well, enabling environments and empowerment, students felt it did not always reflect their realities in practice.

*“I liked the programme, don’t get me wrong, but sometimes the person is really sick. They are really confused, and they are angry…in some cases quite violent and a lot of this doesn’t apply” [FG6, P10, Fifth Year Medical Student].*

*“I am still out of my depth to be fair. The training is brilliant for early stages, but I don’t work with those people, I work with people who are really distressed and have lots of challenging behaviour” [FG3, P1, Third Year Nursing Student].*

Students suggested that future programmes should provide adequate consideration to those with advancing dementia. Participants suggested that content might focus on aspects like ‘truth-telling during diagnosis’, ‘therapeutic lying’, ‘resolving conflict amongst different family members’ and ‘end-of-life symptoms’.

## Discussion

The Alzheimer’s Society ‘dementia friends’ programme has previously been positively evaluated amongst nursing students in Northern Ireland at the time of delivery [28]. To our knowledge, this is the first piece of empirical research which provides a qualitative evaluation of the ‘dementia friends’ programme from the perspective of nursing and medical students in the United Kingdom. A key outcome for this research is that the dementia friends programme reframed the student’s perception of dementia that led to students being more empathetic towards people living with dementia, this aided better communication through verbal and non-verbal techniques. Another key outcome was how this training equipped student healthcare professionals with an understanding of the role the environment plays for people living with dementia and actively supported nursing and medical students in identifying environmental adaptions which ultimately will improve the care and experience for people with dementia.

Many students enter their professional medical professions post-education with a lack of knowledge and negative attitudes about dementia [29] due to the lack of dementia education in their pre-registration education [30]. However, there is the potential for them to learn. The ‘dementia friends’ programme is promising and reflective of other approaches to student dementia education as students often prefer a hands-on approach and activity-centred programmes [31]. This type of programme has improved students’ empathy across the globe from Central South China to Southwest United States [32, 33].

The dementia friends programme educates participants on the early onset stages of dementia which promotes and empowers people living with an early diagnosis of dementia. However, as dementia is a progressive disease those participating often need further education on the advanced stages of dementia which isn’t included in the dementia friends programme, and this is often the stage that healthcare professionals care for the most in an acute setting. While students acknowledged that this education would come later in their professional programme, focus-group data suggested that students were keen to learn more about dementia during this session. The realities of advanced dementia and how to provide effective symptom management later in the disease (i.e., malnutrition, distress, and end-of-life care) were viewed as important given the high possibility of medical and nursing students encountering people with dementia early on in their undergraduate study or clinical placement.

While the ‘dementia friends’ programme has been positively discussed by nursing and medical students in Northern Ireland, there are limitations to its delivery, implementation and sustainability. A potential limitation of this form of education is its design for face-to-face delivery which may not be sustainable in the future. While the ‘dementia friends’ education could be modified for remote delivery via online webinars, it is uncertain whether this would impact the quality of education that learners receive. Having an appropriately qualified trainer to deliver the programme is also a potential barrier to delivering the Alzheimer’s Society dementia friend programme. In response to these challenges, there have been a range of recent asynchronous learning activities about dementia that have been employed to support student understanding of the condition. For example, a dementia awareness game [34, 35] and a dementia photobook [36] have also been used to foster understanding about dementia in recent years. While these interventions lack face-to-face delivery, their positive findings make them viable alternatives to higher education providers that are not able to implement a programme of this nature.

### Strengths and Limitations

A strength of this study was that it is a small interdisciplinary project which explored an innovative approach to dementia education with nursing and medical students. To our knowledge, this is the first time this approach has been explored involving two healthcare professions. This paper highlights shared understanding and the importance of social learning which showcases how a relatively small intervention in healthcare professional’s curricula could leave a significant impact on future healthcare workers with the potential of improving patient outcomes. While this study will make an important contribution to the evidence base, the findings may be difficult to generalise in other universities, as not every university can embed this type of programme in their nursing and medical curriculum therefore it is difficult to replicate. Another limitation of this study is the small sample of participants used for data collection which may not provide an accurate representation of this population. Further, due to the lack of a control group, it is also difficult to determine the extent to which the ‘dementia friends’ education directly impacted medical and nursing student practice. As noted, this education was supplementary, and it is therefore difficult to determine what positive changes are related to ‘dementia friends’ and what are related to pre-existing education.

A further strength of this study is the co-design of the interview guide with people living with dementia. People with a diagnosis are the key experts in this area as they are able to accurately describe this complex disease from their lived experience. This is important for healthcare professionals as they need to be dementia friendly from the perspective of people living with dementia to be able to offer the current support during their care.

## Conclusion

This study adds to the body of evidence supporting the offering of ‘dementia friends programme to all healthcare students. The research highlights the benefits of a two-hour face-to-face session with an Alzheimer’s Society accredited facilitator, in breaking down the stigma often associated with dementia. The programme has a significant impact on how nursing and medical students support people with dementia in their role as medical students or nursing students after receiving this type of education. This fundamentally showcases how a relatively small intervention in health professions curricula can leave a significant impact on future healthcare workers with the potential of improving patient outcomes.

### Electronic supplementary material

Below is the link to the electronic supplementary material.

Supplementary Material 1

## Data Availability

The datasets used and/or analysed during the current study are available from the corresponding author on reasonable request.
