# Supplementary material for: Translating ‘dementia friends’ programme to undergraduate medical and nursing practice: a qualitative exploration
Source: BMC Med Educ. 2023 Aug 7;23:555. doi: 10.1186/s12909-023-04561-1 (PMC10408182; doi:10.1186/s12909-023-04561-1)
Supplement: Supplementary file 1 — Supplementary Material 1 [file 12909_2023_4561_MOESM1_ESM.doc]

**Focus-Group Interview Guide**

**Title of Project:**

**A Qualitative Evaluation of the Dementia Friends Programme delivered to Undergraduate Medical and Nursing Students in Northern Ireland.**

**[All focus-group interviews to be conducted by Dr Gary Mitchell & Miss Stephanie Craig]**

The purpose of this focus-group interview is to gain an understanding of how the ‘dementia friends’ training has impacted your practice when caring for people with dementia throughout your clinical placements. There are a few areas I would like to discuss about your experience. If any participant wishes to stop or withdraw from the interview at any time, please let me know and the interview will be stopped.

***Topic Areas:***

1. *What were your initial impressions about the dementia friends training?*

- Did it change your perception of people living with dementia?
- Did you enjoy the dementia friends training?
- Would you recommend that colleagues on your course continue to receive the training?

1. *How did you apply your dementia friends training when you supported people with dementia on placement?*

- Did you feel more confident communicating with people living with dementia?
- Did you feel more competent in caring with people living with dementia?
- Did you feel more able to empathise with people living with dementia?
- Did you feel more knowledgeable about dementia in general?

1. *How did you apply your dementia friends training when you supported carers/family members of people living with dementia on placement?*

- Did you feel more confident communicating with carers of people living with dementia?
- Did you feel more able to empathise with carers of people living with dementia?
- Did you feel more knowledgeable about the role of carers in dementia care?

1. *Did the ‘dementia friends’ training help you to improve your practice?*

- Did you help people with dementia overcome distress/behavioural problems?
- Did you help adapt the clinical environment to help people with dementia?
- Did you modify your approach to communicating with people with dementia?
- Did you use any creative approaches to supporting people with dementia on placement?
- Did you support colleagues on placement to adapt their practice in any way as a result of the dementia friends training?

*v. What are the limitations of the dementia friends training?*

- How might the ‘dementia friends’ training be improved for your colleagues in the future?
- Did the ‘dementia friends’ training support you in all aspects of your role?
- Has the ‘dementia friends’ training encouraged/discouraged you to think about a career in caring for people living with dementia

Thank you for your time, this has been very helpful and I would be extremely interested in any other thoughts or feelings you have and would like to share to help me better understand your experience or is there anything you would like me to go back to?
